# Physical activity and its correlates in children: a cross-sectional study (the GINIplus & LISAplus studies)

**DOI:** 10.1186/1471-2458-13-349

**Published:** 2013-04-16

**Authors:** Sandra Ortlieb, Gabriel Schneider, Sibylle Koletzko, Dietrich Berdel, Andrea von Berg, Carl-Peter Bauer, Beate Schaaf, Olf Herbarth, Irina Lehmann, Barbara Hoffmann, Joachim Heinrich, Holger Schulz

**Affiliations:** 1 Institute of Epidemiology I, Helmholtz Zentrum München, German Research Center for Environmental Health, Ingolstädter Landstrasse 1, Neuherberg, 85764, Germany; 2 Institut für Medizinische Statistik und Epidemiologie, TUM, Munich, Germany; 3 Dr von Hauner Children’s Hospital, Ludwig-Maximilians-University of Munich, Munich, Germany; 4 Department of Pediatrics, Marien-Hospital Wesel, Wesel, Germany; 5 Department of Pediatrics, Technical University of Munich, Munich, Germany; 6 Medical Practice for Pediatrics, Bad Honnef, Germany; 7 Faculty of Medicine, Environmental Hygiene and Environmental Medicine, University of Leipzig, Leipzig, Germany; 8 Department for Environmental Immunology, Helmholtz Centre for Environmental Research – UFZ, Leipzig, Germany; 9 IUF Leibniz Research Institute for Environmental Medicine and Medical Faculty, Heinrich-Heine University of Düsseldorf, Düsseldorf, Germany

**Keywords:** Activity, Children, Correlates, Exercise, Inactivity, Determinants, Associations, Behavior, Environment, Social

## Abstract

**Background:**

Physical inactivity among children is an increasing problem that adversely affects children’s health. A better understanding of factors which affect physical activity (PA) will help create effective interventions aimed at raising the activity levels of children. This cross-sectional study examined the associations of PA with individual (biological, social, behavioral, psychological) and environmental (East *vs.* West Germany, rural *vs.* urban regions) characteristics in children.

**Methods:**

Information on PA and potential correlates was collected from 1843 girls and 1997 boys using questionnaires during the 10-year follow-up of two prospective birth cohort studies (GINIplus and LISAplus). Study regions represent urban and rural sites as well as East and West of Germany. Logistic regression modeling was applied to examine cross-sectional associations between individual as well as environmental factors and PA levels.

**Results:**

Five of fourteen variables were significantly associated with PA. Among children aged 10, girls tended to be less active than boys, especially with respect to vigorous PA (OR = 0.72 for summer). Children who were not a member of a sports club showed a substantially reduced amount of PA in winter (OR = 0.15). Rural environments promote moderate PA, particularly in winter (OR = 1.88), whereas an increased time outdoors primarily promotes moderate PA in summer (OR = 12.41). Children with abnormal emotional symptoms exhibited reduced physical activity, particularly in winter (OR = 0.60). BMI, puberty, parental BMI, parental education, household income, siblings, TV/PC consumption, and method of arriving school, were not associated with PA.

**Conclusions:**

When considering correlates of PA from several domains simultaneously, only few factors (sex, sports club membership, physical environment, time outdoors, and emotional symptoms) appear to be relevant. Although the causality needs to be ascertained in longitudinal studies, variables which cannot be modified should be used to identify risk groups while modifiable variables, such as sports club activities, may be addressed in intervention programs.

## Background

PA has a positive effect on health and wellbeing at any age. PA during childhood provides beneficial short-term and long-term effects on physical and mental health [1-3], and health behaviors developed during this time may persist into adulthood [4]. Beneficial health effects include improved cardiorespiratory fitness, muscular fitness, bone health, body composition and cardiovascular and metabolic health biomarkers [1-3].

Nevertheless, evidence from cohort studies [5,6] as well as longitudinal studies [7] demonstrate that there is a rapid decline in physical activity (PA) from childhood to adolescence and only one third of European children achieve the current guidelines of at least 60 minutes moderate to vigorous physical activity (MVPA) per day [1,8]. In Germany, 20.4% of children aged 7–10 and only 6.6% of adolescents aged 14 – 17 achieve these recommendations [6]. In so far, the majority of German children do not achieve international recommendations. The disparities in PA participation suggest that some children may not have the possibilities or support they need to be physically active. For targeted interventions it is of high interest to identify factors influencing the PA of children. Different studies on correlates of PA among children indicate that PA is influenced by multidimensional aspects including biological, social, behavioral, psychological, and environmental factors [9,10]: Males are more active than females, and these differences extant into adulthood [9,11,12]. Conflicting results emerge from studies on the association between socioeconomic conditions and level of PA in young people [11,13]. There is also disagreement regarding sedentary habits, like watching TV, and their relationship to PA levels in adolescents. An increasing amount of time spent in sedentary activities was found to reduce PA [14]. On the other hand, there is evidence that sedentary activities are not automatically a barrier for children to engage in the recommended amounts of PA [15]. Physical activity has been associated consistently with better psychological health, especially higher levels of self-efficacy [16,17] and findings from socio ecological studies have shown a positive relationship between environmental factors, such as family [18] or school support [19], and PA.

Although many factors have been individually investigated in separate studies, no study was found which considered all factors mentioned above in children aged ten simultaneously. The consideration of only one or a few potential correlate may lead to an overestimation of the association between PA and the relevant factor. Therefore, the present study aims to fill this gap by examining multiple factors simultaneously. The objective of the present study is to assess the seasonal relationship between the levels of PA and potential factors that induce, facilitate and strengthen the practice of PA in children. Our study is based on data from the 10-year old follow-up of two large population-based birth cohort studies originating from four regions in Germany [20-24]. The correlates are assigned according to their origin to one of the following groups: biological, social, behavioral, psychological, and environmental.

## Methods

### Study population

The present analysis is based on two ongoing German birth cohorts. GINIplus (German Infant Study on the influence of Nutrition Intervention plus environmental and genetic influences on allergy development) is a prospective cohort study originally designed to investigate the influences of a nutrition intervention during infancy, air pollution and genetics on allergy development. Details of the study design, recruitment, and follow-up have been previously described [23,24]. In brief, between September 1995 and June 1998, a total of 5991 term newborns were recruited from two regions in Germany (Munich and Wesel). All candidate mothers in these regions were contacted before or shortly after delivery and were requested to answer a questionnaire with 18 items on past or present asthma, allergic rhinitis, atopic dermatitis, allergic urticaria, or food allergy. Parents with a healthy newborn and at least one family member with an allergic disease pursuant to the questionnaire were asked to participate. All included infants were followed up annually from birth to six years and at 10 years of age. Information on health outcomes and covariates, such as nutrition and physical activity, were collected using parent-completed questionnaires. A total of 3317 children (55.4% of the original study population) participated in the 10-year follow-up. Loss to follow-up was associated with a lower level of parental education, a lack of parental atopy, the absence of atopic diseases in the child during the first 2 years of life and residency in Wesel. The presence of older siblings was not associated with study discontinuation [25].

LISAplus is a population-based birth cohort study initiated to assess the “influence of lifestyle-related factors on the immune system and the development of allergies in childhood”. Between November 1997 and January 1999, a total of 3095 newborns were finally recruited from four German cities: Munich, Leipzig, Wesel and Bad Honnef. Neonates presenting at least one of the following criteria were excluded from the study: preterm birth (maturity < 37 gestational weeks), low birth weight (< 2500 g), congenital malformation, symptomatic neonatal infection, antibiotic medication, hospitalization or intensive medical care during neonatal period. Moreover, newborns from mothers with immune related diseases, on long-term medication or who abuse drugs and/or alcohol, and newborns from parents with a nationality other than German or who were not born in Germany, were not included. Further information about the design and objective of this study are described elsewhere [20,21]. Data on family history of atopy, parental education, physical activity and other lifestyle factors were obtained using parent-completed questionnaires at regular time intervals (0.5, 1, 1.5, 2, 4, 6 and 10 years of age). A total of 1761 children (56.9% of the original study population) participated in the 10-year follow-up. Loss to follow-up was associated with a lower level of parental education and residency in Wesel or Leipzig, whereas the absence of older siblings, a lack of parental atopy and absence of childhood atopic diseases during the first 2 years of life showed a weak association with study discontinuation [25].

All questionnaires were administered 4 weeks before the 10th birthday of each child for the 10-year-follow-up. Altogether, 5078 (55.9%) parents completed and returned the questionnaires. To be included in this cross-sectional study, all six questions about physical activity had to be answered, which decreased the available sample size to 3840 subjects. Due to missing values in the other variables of interest, the study population was further reduced to the final number of 2809 subjects for the multivariate analysis.

To justify the pooling of data from both cohorts originating from four study regions, distributions of each variable were compared by cohort *a priori*. The *t*-test was applied for continuous variables with normal distribution, the Wilcoxon-Mann-Whitney-Test for continuous variables that were not normal distributed, and the Chi-Quadrat-Test of Pearson was used for categorical variables.

Both study protocols were approved by the local ethnics committees (Bavarian General Medical Council, University of Leipzig, Medical Council of North-Rhine-Westphalia) and written informed consent was obtained from all participating families.

### Outcome

#### Physical activity

PA was assessed by questionnaire. The questions regarding PA were based on a questionnaire from a huge German cohort study, called German Health Interview and Examination Survey for Children and Adolescents (KiGGS), which offers nationwide representative data on the motor fitness and physical activity of children and adolescents. The questionnaire shows good overall test-retest reliability (r = 0.97) and good validity for questions related to PA (r = 0.26-0.66) [26]. Parents had to answer six questions about the time their child spent in each of three activity levels per week (see Additional file 1, extract of the GINI 10 questionnaire). A characterization of the activity levels was given to the parents in order to facilitate the appraisal. Categories included light activity which was defined as “no sweating, normal respiration (e.g. walking)”, moderate activity which indicated “some sweating, moderately increased respiration (e.g. cycling, swimming, skating)” and vigorous activity which was defined as “strong sweating, fast respiration (e.g. ball games, training)”. Since there is evidence that PA is higher in summer than in other seasons [27], PA was requested separately for winter and summer. As a result there were six distributions (light, moderate, and vigorous PA, for winter and summer each) appropriate to the activity hours which were divided on the basis of their respective quartiles. Each distribution was divided into 3 categories: one group included children in the lowest quartile (“rare”: < 25th percentile), a second group those in the two intermediate quartiles (“average”: ≥ 25th – < 75th percentile) and a third group contained children in the highest quartile (“frequent”: ≥75th percentile). The categorical classification mentioned above was used to minimize the probability of bias resulting from questionnaires.

### Definition of correlates

All measured variables reported in the 10-year follow-up which may be associated with PA were included in the study. How each variable was defined for this study is described below. For the correlates, a five-category classification system was used [9]: biological factors (sex, BMI, status of puberty, siblings, parental BMI), social factors of the parents (education and income), behavioral factors (TV and computer habits, method of arriving to school, sports club membership, time outdoors), physical environmental factors (neighborhood), and psychological factors (emotional symptoms).

### Biological factors

#### Height and weight

Height and weight of the children were measured during the 10-year medical examination (66.6% of participants). In case the children were not examined, data from the questionnaire (33.4% of participants) were used.

#### Body mass index (BMI)

BMI was calculated using the height and weight information. BMI was classified according to the percentile graphs of Kromeyer-Hauschild, as recommended for German children and adolescents [18]: Children with BMI < 10 percentile were classified as underweight, ≥ 10 to < 90 percentile as normal, ≥ 90 to < 97 percentile as overweight and ≥ 97 percentile as obese. For more information, see Kromeyer-Hauschild [28].

#### Status of puberty

Status of puberty was assessed using the following question: Does your child show characteristics which indicate puberty (yes/no?)” to identify if puberty was already reached or not. Based on the questionnaire “Pubertal Development Scale [29] different characteristics were given as representative of start of puberty: aces, pimples; pubic/axillary hair; breast development; menstruation; penis/testes enlargement; others.

#### Siblings

Information regarding number of siblings was collected by questionnaire. For this analysis, these data were dichotomized (yes/no).

#### Parental BMI

The BMI of the parents was calculated using values of height and weight obtained by questionnaire. According to WHO (World Health Organization) criteria [30], the parents were classified as underweight (BMI < 18.5 kg/m^2^), normal weight (BMI ≥ 18.5 and < 25 kg/m^2^), overweight (BMI ≥ 25 and < 30 kg/m^2^), and obese (BMI ≥ 30 kg/m^2^). After conducting descriptive analyses, underweight and normal weight categories were combined due to the small number of subjects in the underweight group.

### Social factors of the parents

#### Parental education

Parental education was classified into three categories based on the highest grade completed by either the father or the mother. Hence, children were assigned to low (less than tenth grade), medium (tenth grade) or high (more than tenth grade) parental education [25].

#### Household income

Parents were asked to indicate their household income using nine categories which ranked from < 500 € to > 3500 €. To compare different study areas and different household sizes, an equivalent income was calculated according to the guidelines of the OECD (Organisation for Economic Cooperation and Development) [31]. In order to get a comparable class size, the household income was organized into three categories (low, medium and high). Further details on the derivation of the parental education and household income variables, which represent the socio-economic status, are provided in two studies of Sausenthaler et al. [25,32].

### Behavioral factors

#### Consumption of TV and PC

There were four answers possible for the question pertaining to TV and personal computer (PC) use. However, the categories “3-4 hours a day” and “more than 5 hours a day” were merged because of their low response frequency. Consequently, the variable TV and PC use consisted of 3 categories: “<1 h per day“, “1-2 h per day“, and “> 2h per day“. As other studies found that sedentary behavior was lower in summer than in other seasons [33,34], information for the summer and winter seasons was requested separately.

#### Method of arriving at school

Based on the German KiGGS study [26], the variable “method of arriving at school” had four possible answers: “on foot”, “by bike”, “by bus/train” or “by car”. Here, multiple choices were included. For the analyses, the four categories were reduced to “active” (“on foot” or “by bike”), “passive” (“by bus/train” or “by car”) or “alternate” (sometimes “active”, sometimes “passive”).

#### Sports club membership

The questionnaire comprised one question regarding sports club membership and one regarding the type of sport, as other studies did [26]. For the present analysis, only the information regarding the sports club membership was considered.

#### Time outdoors

In order to capture the time children spent outdoors, the question “How many hours is your child outside on an ordinary working day” was asked, similar to the huge German KiGGS study [26]. Parents could choose between three answers for both summer (“<3 h“, “3-5 h“or “>5 h“per day) and winter (“<2 h“, “2-3 h“and “>3 h“per day).

### Environmental factors

#### Seasons

Questions of PA were asked separately for winter and summer in order to detect differences in activity habits between seasons [27]. TV and PC consumption as well as time outdoors were also examined by season [33,34].

#### Neighborhood

The participants were from four cities in Germany: Munich, Leipzig, Bad Honnef and Wesel. This sample allows comparisons between urban (Munich and Leipzig) and rural areas (Bad Honnef and the region of Wesel). The Wesel region encompasses 37 adjacent communities inhabited by between 8000–80.000 people each, and covers approximately 3300 km^2^[22]. Moreover this sample of cities also offers the possibility to make comparisons between East (Munich) and West (Leipzig) Germany.

### Psychological factors

#### Emotional symptoms

To detect emotional symptoms of the children, the German version of the Strengths and Difficulties Questionnaire (SDQ) was completed by the parents. The SDQ is a screening questionnaire established to detect behavioral strengths and difficulties of children 3 to 16 years old and shows good test-retest reliability (mean Cronbach α: 0.62) as well as a good validity [35]. For example, Goodman et al. [36] identified a highly significant level of chance-corrected agreement between SDQ prediction and an independent clinical diagnosis (Kendall’s tau b between 0.49 and 0.73; p < 0.001). “Emotional symptoms” is one of 5 subscales and consists of 5 items ((1)“Often complaints of headaches, stomach-aches or sickness”, (2) “Many worries, often seems worried”, (3) “Often unhappy, down-hearted or tearful”, (4) “Nervous or clingy in new situations, easily loses confidence, (5) “Many fears, easily scared”) with 3 response options (“not true” (scored as 0), “somewhat true” (scored as 1), “certainly true” (scored as 2)). The cut points of the German validation [37] were chosen to classify approximately 10% of children as “borderline” and 10% as “abnormal.” Based on these cut off points, the total score is classified as “normal” (scored 0–3), “borderline” (scored 4), or “abnormal” (scored 5–10).

### Statistical analysis

Statistical analyses were carried out using SAS version 9.2. To compare the two cohorts, GINI and LISA, several tests were performed: we used a q-q plot as well as the Independent Samples *t*-test for anthropometric data of the children and their parents, to compare the categorical factors we used Chi-Quadrat test, and for comparison of the PA distributions of GINI and LISA (stratified by activity level in summer and winter each) we used the Mann–Whitney *U*-test. For comparisons between non-responders and responders the same tests were used. After stratification by sex and study region, the Kruskal-Wallis test was used for continuous factors and the Pearson Chi-Quadrat test was used for categorical variables. Values of continuous factors are provided as mean and standard deviation (SD) and those of categorical factors are reported as percentage (%) of the respective variable. An alpha level of 0.05 was used to indicate statistical significance. Although our original intention was to conduct the analysis using the ordinal logistic regression model to make optimal use of the three categories contained within the dependent PA variable, this could not be realized because the proportional odds assumption was not valid. Therefore, nominal regression models were calculated, which use one of the activity categories as the reference category and evaluate two independent estimates for the other categories. For this analysis, “low activity” was used as the reference category. Altogether, this results four regression models: moderate and vigorous physical activity in summer and winter, as shown in Table 1. Backward elimination was used to select the variables for the models. Associations are reported as adjusted odds ratios (OR) and 95% confidence intervals (CI). The adjusted nominal regression analyses were operated as a complete case analysis. Subjects with more than 10% missing values were eliminated. To assess a potential selection bias characteristics of subjects not included in the adjusted analyses due to missing values were compared with subjects included in the model by the Pearson Chi-Quadrat test. Moreover, single imputation was used to fill in missing data in the dataset and finally to compare the results with those of the complete case analysis.

**Table 1:** Dependent and independent variables of the four nominal regression models

**Dependent variables**	Moderate PA in summer
	Moderate PA in winter
	Vigorous PA in summer
	Vigorous PA in winter
**Independent variables**	*Biological*
	Sex
	BMI
	Puberty
	Siblings
	BMI of mother
	BMI of father
	*Social*
	Parental education
	Parental income
	*Behavioral*
	TV/PC consumpion
	Method of arriving at school
	Sports club membership
	time outdoors
	*Environmental*
	Neighborhood
	*Psychological*
	Emotional symptoms

## Results

### Descriptive results

No notable differences in the variable distributions were noted between the two cohorts, GINI and LISA, thus the data was pooled. This also applies to responders vs. non-responders. Moreover, no differences were found between the entire study population (n = 3840) and those subjects included in the nominal regression model (n = 2809) due to complete-case analysis.

The study population included 3840 children (1997 male and 1843 female), who had a mean height of 143.9 cm (± 6.6), a mean weight of 35.8 kg (± 6.7), and a mean age of 10.1 (± 0.24). Of those who participated, 54.0% and 8.3% came from the two urban German regions Munich and Leipzig, respectively, and 4.2% and 33.5% originated from the more rural regions Bad Honnef and Wesel, respectively. In summer, the mean times per week that children passed in the three different activity levels were 12.7 h (± 11.0) for light activity, 8.2 h (± 6.1) for moderate activity, and 5.5 h (± 4.5) for vigorous activity. Appropriate mean times in winter were 9.5 h (± 8.9) for light activity, 5.2 h (± 4.3) for moderate activity, and 5.0 h (± 3.3) for vigorous activity.

Table 2 records the characteristics of each variable stratified by sex. There were clear differences with respect to moderate and vigorous PA. Girls were less active than boys, especially for vigorous PA in summer as well as in winter. No differences across genders were observed for child BMI, siblings, parental education, equivalent income, parental BMI, method of arriving at school, and emotional symptoms. In contrast, the variables puberty status (12.2% of boys vs. 49.0% of girls showed indications of puberty), TV and PC consumption, membership of a sports club and time outdoors in winter were significantly different between males and females.

**Table 2:** Characteristics of the study population: school children (10 years), stratified by sex

			**Total (n 3840)**	**Boys (n 1997)**	**Girls (n 1843)**	
			**%**	**%**	**%**	p-Value^*^
***Outcome***						
**Physical activity**^**a **^**(per week)**						
*Summer*	*Light*	< 6 h	26.3	27.6	24.9	0.0569
		6 – 14 h	44.7	44.8	44.6	
		> 14 h	29.0	27.6	30.6	
	*Moderate*	< 5 h	30.3	28.4	32.5	0.0113
		5 – 9 h	34.5	34.7	34.4	
		> 9 h	35.1	36.9	33.2	
	*Vigorous*	< 4 h	36.5	26.2	47.5	<.0001
		4 – 6 h	35.3	37.8	32.7	
		> 6 h	28.2	36.0	19.8	
*Winter*	*Light*	< 5 h	28.4	28.7	28.1	0.8874
		5 – 11 h	45.8	45.7	45.9	
		> 11 h	25.8	25.6	26.1	
	*Moderate*	< 3 h	28.5	26.9	30.3	0.0175
		3 – 6 h	42.6	42.5	42.6	
		> 6 h	28.9	30.6	27.1	
	*Vigorous*	< 3 h	33.8	27.7	40.4	<.0001
		3 – 4 h	33.2	32.6	33.8	
		> 4 h	33.1	39.7	25.9	
***Biological***						
**BMI**^**b**^		Underweight	8.7	8.7	8.7	0.6698
		Normal weight	82.3	81.9	82.8	
		Overweight	6.6	6.7	6.5	
		Obese	2.4	2.7	2.1	
**Start of puberty**		yes	29.9	12.2	49.0	<.0001
**Siblings**		yes	87.9	87.7	88.1	0.6928
**Parental BMI**^**c**^	*Mother*	Underweight	2.5	2.4	2.6	0.4895
		Normal weight	70.0	69.7	70.3	
		Overweight	19.6	20.4	18.8	
		Obese	7.9	7.4	8.3	
	*Father*	Underweight	0.2	0.3	0.1	0.2545
		Normal weight	43.6	44.3	42.8	
		Overweight	45.2	44.6	46.0	
		Obese	11.0	10.8	11.2	
***Social***						
**Parental education**^**d**^		Low	5.9	6.5	5.3	0.2067
		Moderate	26.0	26.5	25.4	
		High	68.1	67.0	69.3	
**Household income**^**e**^		Low	6.4	6.7	6.1	0.2522
		Moderate	22.7	23.6	21.7	
		High	70.9	69.7	72.2	
***Behavioral***						
**TV/PC (per day)**	*Summer*	< 1 h	68.0	64.3	72.0	<.0001
		1 – 2 h	30.4	33.6	26.8	
		> 2 h	1.6	2.1	1.2	
	*Winter*	< 1 h	32.6	29.6	35.8	<.0001
		1 – 2 h	56.4	57.6	55.1	
		> 2 h	11.0	12.8	9.1	
**Method of arriving school**^**f**^		Active	65.6	66.6	64.6	0.3355
		Passive	26.6	25.6	27.7	
		Mixed	7.8	7.8	7.8	
**Membership of a sports club**		yes	85.1	86.8	83.3	0.0021
**Time Outdoors (h per day)**						
	*Summer*	< 3 h	11.5	10.7	12.4	0.2373
		3 – 5 h	68.4	69.3	67.4	
		> 5 h	20.1	20.0	20.2	
	*Winter*	< 2 h	30.3	27.6	33.2	0.0002
		2 – 3 h	60.7	62.2	59.0	
		> 3 h	9.1	10.2	7.8	
***Psychological***						
**Emotional Symptoms**^**g**^		Normal	82.7	83.2	83.2	0.3519
		Borderline	7.4	6.8	8.1	
		Abnormal	9.8	10.0	9.7	

^*^p-values from Pearsons Chi-Quadrat Test; ^a^Physical activity: light activity (no sweating. normal respiration. like walking). moderate activity (some sweating. moderately increased respiration. like cycling/swimming) and vigorous activity (strong sweating. fast respiration like ball games/training); ^b^BMI according to the classification by Kromeyer-Hauschild et al.; ^c^Parental BMI according to WHO classification; ^d^Parental education: low (less than tenth grade). moderate (tenth grade). high (more than tenth grade); ^e^Household income adjusted for size of household and study region (class size based on distribution of parternal education); ^f^How they reach school: active (by foot or by bike). passive (by car. bus or train). mixed (alternate active and passive way to school); ^g^Emotional symptoms according to the German version of the Strengths and Difficulties Questionnaire.

Table 3 shows the variables stratified by study region. When considering the study regions significant differences were detected for all PA levels in summer and winter but the overall data is not consistent. In summer, the percentage of children engaged in long periods of moderate and vigorous PA (> 9 h and > 6 h, respectively) appears to be somewhat lower in the urban regions, Munich and Leipzig, compared to the rural sites, Wesel and Bad Honnef. This holds also true for more than 6 h moderate PA in summer, but values for more than 4 h vigorous PA in winter were only lower in Munich compared to the three other sites which showed comparable values. There were no significant differences in sex, emotional symptoms, puberty status and equivalent income among study regions. In contrast, all the other biological, social, and behavioral variables showed highly significant differences between the four study regions. Children from rural regions show higher BMI values, longer periods of time outdoors as well as of total PA, and seem to have more siblings. There were also appreciable differences between the study regions with respect to parental education and PC/TV consumption. Parental education was lowest in Wesel (51.1%) and highest in Munich (79.2%). Moreover, the TV/PC consumption rate in winter was nearly three times higher in Wesel than in Munich with respect to consumption of more than two hours.

**Table 3:** Characteristics of the study population: school children (10 years), stratified by study region

			**Munich (n 2075)**	**Leipzig (n 319)**	**Bad Honnef (n 161)**	**Wesel (n 1285)**	
			**%**	**%**	**%**	**%**	p-Value^*^
**Physical activity**^**a **^**(per week)**							
*Summer*	*Light*	< 6 h	30.4	24.8	24.2	20.4	<.0001
		6-14 h	46.3	44.5	46.6	41.9	
		> 14 h	23.3	30.7	29.2	37.7	
	*Moderate*	< 5 h	34.9	29.5	32.9	22.8	<.0001
		5-9 h	34.8	40.1	28.0	33.5	
		> 9 h	30.2	30.4	39.1	43.7	
	*Vigorous*	< 4 h	39.3	37.3	38.5	31.4	0.0003
		4-6 h	34.4	35.4	29.8	37.6	
		> 6 h	26.3	27.3	31.7	31.1	
*Winter*	*Light*	< 5 h	31.8	24.8	29.8	23.6	<.0001
		5-11 h	47.4	48.0	44.7	42.8	
		> 11 h	20.8	27.3	25.5	33.6	
	*Moderate*	< 3 h	33.0	26.3	29.8	21.7	<.0001
		3-6 h	42.6	48.9	41.6	41.0	
		> 6 h	24.4	24.8	28.6	37.3	
	*Vigorous*	< 3 h	38.0	26.7	32.3	28.9	<.0001
		3-4 h	32.5	36.4	29.8	33.9	
		> 4 h	29.5	37.0	37.9	37.3	
***Biological***							
**Sex**		Boys	52.3	52.4	50.9	51.5	0.9593
**BMI**^**b**^		Underweight	9.9	6.6	7.1	7.4	<.0001
		Normal weight	83.4	86.6	78.7	80.0	
		Overweight	4.9	4.9	10.3	9.3	
		Obese	1.8	2.0	3.9	3.3	
**Start of puberty**		Yes	30.3	34.7	31.5	27.7	0.0784
**Siblings**		Yes	86.6	82.8	89.4	91.1	<.0001
**Parental BMI**^**c**^	*Mother*	Underweight	3.1	2.4	3.9	1.5	<.0001
		Normal weight	72.9	65.8	64.7	67.1	
		Overweight	17.4	22.4	21.6	22.3	
		Obese	6.7	9.5	9.8	9.2	
	*Father*	Underweight	0.3	0.0	0.0	0.2	<.0001
		Normal weight	47.5	45.0	43.2	37.1	
		Overweight	43.9	43.5	45.3	47.7	
		Obese	8.3	11.4	11.5	15.0	
***Social***							
**Paternal education**^**d**^		Low	3.7	1.3	3.8	11.0	<.0001
		Middle	17.2	36.6	21.9	37.9	
		High	79.2	62.2	74.4	51.1	
**Household income**^**e**^		Low	6.7	4.6	5.7	6.4	0.0667
		Middle	23.4	22.0	31.2	20.5	
		High	69.9	73.4	63.1	73.0	
***Behavioral***							
**TV/PC (per day)**	*Summer*	< 1 h	74.9	64.3	67.9	57.9	<.0001
		1-2 h	23.9	32.6	29.6	40.3	
		> 2 h	1.2	3.1	2.5	1.9	
	*Winter*	< 1 h	43.7	30.9	22.6	16.4	<.0001
		1-2 h	50.1	53.6	66.7	66.0	
		> 2 h	6.3	15.5	10.7	17.6	
**Method of arriving school**^**f**^		Active	67.9	47.3	50.6	68.5	<.0001
		Passive	25.1	43.9	38.1	23.2	
		Mixed	7.0	8.8	11.3	8.3	
**Membership of a sports club**		Yes	85.9	76.3	89.4	85.6	<.0001
**Time Outdoors (h per day)**							
	*Summer*	< 3 h	15.8	13.9	5.6	4.7	<.0001
		3-5 h	70.9	70.0	67.5	64.0	
		> 5 h	13.3	16.1	26.9	31.3	
	*Winter*	< 2 h	36.4	31.0	28.3	20.5	<.0001
		2-3 h	57.9	60.2	63.5	64.8	
		> 3 h	5.7	8.8	8.2	14.7	
***Psychological***							
**Emotional Symptoms**^**g**^		Normal	82.6	82.7	85.4	82.7	0.4985
		Borderline	6.9	8.2	5.7	8.4	
		Abnormal	10.5	9.2	8.9	9.0	

^*^p-values from Pearsons Chi-Quadrat Test; ^a^Physical activity: light activity (no sweating. normal respiration. like walking). moderate activity (some sweating. moderately increased respiration. like cycling/swimming) and vigorous activity (strong sweating. fast respiration like ball games/training); ^b^BMI according to the classification by Kromeyer-Hauschild et al.; ^c^Parental BMI according to WHO classification; ^d^Parental education: low (less than tenth grade). moderate (tenth grade). high (more than tenth grade); ^e^Household income adjusted for size of household and study region (class size based on distribution of parternal education); ^f^How they reach school: active (by foot or by bike). passive (by car. bus or train). mixed (alternate active and passive way to school); ^g^Emotional symptoms according to the German version of the Strengths and Difficulties Questionnaire.

### Multivariate results

For each of the four dependent variables moderate and vigorous PA in summer and winter was considered separately by a nominal logistic model. The complete case analysis provided the same results as a single imputation. All the findings described below derive from the complete case analysis.

**Figure 1:**
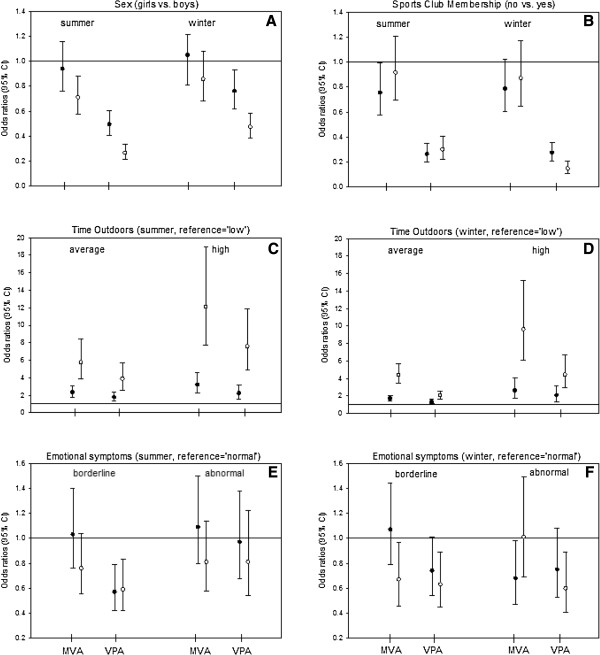
**Results of the four nominal regression models (moderate and vigorous physical activity in summer and winter).** Significant correlates of physical activity I: Odds Ratios (95% CI) for sex (**A**), sports club membership (**B**), outdoor activity in summer (**C**) and winter (**D**), emotional symptoms in summer (**E**) and winter (**F**). MPA = moderate physical activity, VPA = vigorous physical activity.

**Figure 2:**
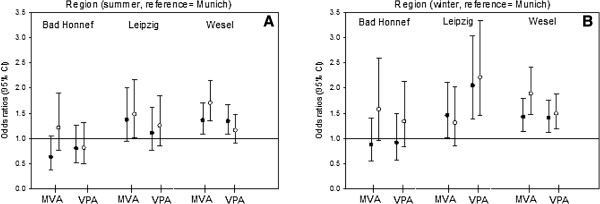
**Results of the four nominal regression models (moderate and vigorous physical activity in summer and winter).** Significant correlates of physical activity II: Odds Ratios (95% CI) for study region in summer (**A**) and winter (**B**). MPA = moderate physical activity, VPA = vigorous physical activity.

There were significant results for sex, neighborhood, sports club membership, time outdoors and emotional symptoms (Figures 1 and Figure 2). These variables appear to be in a strong relationship with the behavior of the activity of the children. Only few other variables had significant results in the four models. There were no correlations between PA and the other variables examined (see Additional file 2: Table S1, results of the four nominal regression models).

Sex was correlated with PA, especially with vigorous PA. Girls showed a lower chance of vigorous PA than boys in both winter and summer. For moderate activity, this effect seems to exist as well, but not so distinctively: only one of the four effect estimates was significant. The influence of sex was stronger in summer than in winter (Figure 1A). A relationship between PA and sports club membership was only observed for vigorous activity. Opportunities to spend time doing vigorous PA was reduced for children who were not a member of a sports club, regardless of season (Figure 1B). Time outdoors was also positively correlated with PA. Again, there was a stronger association between the two variables in summer compared to winter, and the effect was greater for moderate activity levels than for vigorous activity levels. Because of its high odds ratio values, time outdoors seems to be the most relevant correlate for PA in our study (Figure 1C + D). An inverse relationship between PA and emotional symptoms can be observed, especially for vigorous activity. The opportunity to engage in vigorous PA is reduced for children who show borderline or abnormal emotional wellbeing. They tended to have fewer opportunities to engage in moderate PA, but this effect is only significant in winter (Figure 1F + G). Regional differences show similar results for winter and summer. However, the effect seems more pronounced in winter than in summer, which is reflected in the stronger point estimates and the higher number of significant results. Children from Leipzig and Wesel had a greater opportunity to engage in long periods of moderate and vigorous PA than those from Munich. In contrast, living in Bad Honnef does not show a significantly statistical effect on the extent of moderate and vigorous PA (Figure 2A + B).

## Discussion

The present study examined parent-reported PA in different activity levels and the associations with individual and environmental characteristics in children. The mean times that children passed in these activity levels were 57 min/day (6.7 h/week) for moderate PA, and 45 min/day (5.3 h/week) for vigorous PA. The HELENA study reported 71 min/day for moderate and 32 min/day for vigorous PA for self-reported PA across European adolescents [5]. These results are relatively high compared with the current recommendations of 60 min moderate to vigorous PA per day and those reported by objective measures [38]. However, these findings are not surprising because overestimation by questionnaires is a common problem due to recall bias, over-reporting, or by social desirability [5], particularly in young children [39,40]. Accelerometers would have been more suitable to record activity continuously and in more detail. They are supposed to be more accurate instruments to measure the spontaneity and variety of the child’s activity [41]. However, this method was too expensive to implement in the present study and therefore questionnaires were used, as usual for large community-based studies [1,5,6,8].

It has been hypothesized that PA in children is determined by a complex interplay of multiple aspects [42]. Using a multidimensional approach, the present findings suggest that male sex, sports club membership, rural region of residence, increased time outdoors and a normal emotional status are associated with a higher level of PA in children aged 10.

Sallis et al. [9] and van der Horst et al. [10] reviewed studies on correlates of PA in children and adolescence and showed a positive association between sex (male) and children’s PA in almost all of the 36 studies they examined, which is consistent with the finding of the present study. The reasons for sex-specific differences were discussed from different perspectives: while lots of investigators have focused on socialization in sports or exercise involving family, school or peer groups [43,44], a study by Vilhjalmsson et al. [12] found lower enrollment in organized sport clubs among girls and suggested that organized sport may be a key explanatory factor. In the present study, the opportunity to engage in vigorous PA was reduced for children who were not enrolled in a sports club. We also observed a reduced enrollment in sports clubs for girls. Both of our findings provide some support for the hypothesis of Vilhjalmsson et al. However, the study design of the present study does not allow confirming this hypothesis and thus it is important to consider further potential explanatory factors like child’s physical environment. With respect to this factor time spent outdoors was positively correlated with a child’s overall PA. This finding is consistent with the results of Sallis et al. Moreover, the present study demonstrates that the effect of time spent outdoors is most significant for moderate activity levels in summer. The seasonal differences can be ascribed to the nicer weather in Germany during the summer season and agrees with the findings of Conrad et al. [45]. The present findings show that children spent much more time outdoors in summer than in winter and that there is a significant difference in the time spent outdoors between girls and boys, but only in winter.

A neighborhood environment that offers possibilities to be physically active on playgrounds was found to promote PA time among children [42,45,46]. Time spent outdoors decreases with increasing community size and urbanization and is higher in neighborhoods consisting of detached houses [45]. In the present study, four study regions were examined which allow comparisons between urban (Munich and Leipzig) and rural areas (Bad Honnef and Wesel), as well as between East (Leipzig) and West (Munich) Germany. Children from Wesel had a greater chance to be engaged in moderate or vigorous activity than children from Munich. Accordingly, children from more rural areas seem to be more active than those living in urban areas, a finding also often observed by other studies [45,47]. A reason for the increased moderate PA of children from rural regions could be a higher time spent outdoors, as observed among children from Wesel. A comparison between Bad Honnef and Munich did not yield conclusive findings. This is probably caused by the low number of subjects (n = 161) living in Bad Honnef. Differences between East and West Germany, i.e. between Leipzig and Munich, became apparent in winter, especially for high activity levels suggesting that children living in urban areas of East Germany seem to be more active than those in West Germany. Since the climatic conditions between the four study regions are comparable [48] differences cannot be related to the regional climate. Up to now, studies on psychological factors have demonstrated that PA is positively associated with self-esteem and self-concept [49]. With respect to emotional symptoms an inverse association between PA and depression, anxiety, and shyness was found in children [22,50,51]. The present findings support these associations, showing that it plays a more prominent role for high intensity levels of PA. Children with abnormal emotional symptoms probably avoid situations that need “overcoming”, for example, when performing high intensity sports. Anxious or under-confident children are probably less prone to take part in sport class, which we found to be correlated with vigorous PA. Therefore, it would be commendable to enhance childrens’ perceived competence in relation to PA by helping them to overcome the barriers and to go through a series of successful experiences regarding PA [52].

Some variables, for which a link to children’s PA was hypothesized, showed no association. For example, none of the socioeconomic variables contributed to the regression models. Parental education and parental income did not show any effect, except for one subgroup of vigorous PA that was moderately influenced by income. Many other studies have also reported inconsistent associations for parental education and income with PA [9,10,53]. However, comparison of findings with other studies is hampered by the fact that different measures for socio-economic status have been applied, including education, income or occupation [54]. There was also no correlation between parental BMI and PA in the present study. As previously reported [50], no decisive links were observed between the children's BMI and status of puberty and PA. Obesity and overweight were low in the study population, 2.4% and 6.6%, respectively, which may limit conclusions on this subject. Other studies revealed inconsistencies between BMI and PA but the majority could not show an association between the two variables [10]. The same applies to studies which examined the association between puberty and PA. Some studies found PA levels varying with pubertal status whereas other studies could not find any association between puberty and PA [55]. Furthermore, behavioral aspects such as TV/computer consumption or the method by which children reach school did not influence the multivariate model. Indeed, in our study, only few children showed an extreme TV/PC consumption of more than two hours per day and, therefore, it is difficult to draw conclusions on this subject. However, it is remarkable that boys watched more television than girls, but at the same time exhibited higher activity levels. Conflicting results emerge from studies on TV/computer consumption in children suggesting on one hand that increasing amount of time in sedentary activities seems to negatively influence PA [56] while on the other hand, sedentary time is not necessarily an indicator for reduced amounts of PA [15,57,58]. The study by Pate et al. [17] showed that children spending more than three hours of TV/computer consumption per day are more likely to be low active suggesting that there may be a threshold beyond which the impact of TV watching is perceptible on PA behavior [58].

The results of the present study suggest that physically active children show particular characteristics that significantly differ from low active children. Active children are usually a member of a sports club, spend a lot of time outdoors and do not appear to have emotional symptoms such as fear, unhappiness or frequently headaches. Moreover, they tend to be male and live in rural areas. Though these two factors are not modifiable, they can be used to identify children at risk. Preventive interventions should focus on motivating children to increase their PA with particular consideration for girls, children in poor emotional mood and urban children.

Overall our findings are confirmatory but, in contrast to other studies, in the present survey multiple factors associated with PA in children were considered simultaneously which results in fewer significant associations than did other strategies such as bivariate analysis [10]. Hence, the results of the present study exclusively reflect essential correlates of PA in children. The large sample size is another important strength of this study, as it allows the calculation of precise estimates for several different parameters. Moreover, for the multivariate analysis, categorical data were used which rendered our results less susceptible to outliers. Limitations of this study include the reliance on parent-reported data and the cross-sectional study design. Moreover, there may be additional correlates of PA which were not measured and therefore could not be considered in our study, like family support, parental PA or school-based strategies that encompass physical education, classroom activities, and after-school sports [9,10,46]. Interpretations according to the socioeconomic status should be treated with caution as the socioeconomic status of the ten year follow up is above average. There is evidence that PA questionnaires lead to higher levels of self-reported physical activity than measuring it with the help of accelerometers [38]. However, because of the large sample size of our study questionnaires were more suitable. Nevertheless, there is need to determine a valid and inexpensive instrument that can be used in large community-based studies to collect physical activity behavior of children. Furthermore, it is essential to initiate longitudinal studies to identify the direction of causality of potential correlates of PA.

## Conclusions

It is essential to consider variables of different domains in order to improve our understanding of important factors which influence PA during childhood. This will allow the identification of children in need and the implementation of effective PA promotion strategies. Considering correlates of physical activity from several domains simultaneously, the present findings suggest that male sex, sports club membership, rural region of residence, increased outdoor time and normal emotional status are associated with a higher level of PA in children. It is important to consider all potential correlates in order to understand the context of physical activity. Variables which cannot be modified can be used to identify risk groups while modifiable variables, such as sports club activities, should be addressed by intervention programs as far as the causality has been clarified.

## Competing interest

The authors declare that they have no competing interests.

## Authors’ contributions

In this German multicenter study SO, GS, and HS wrote the manuscript and were responsible for data analysis and interpretation as well as statistical expertise. SK, DB, AB, CPB, BS, OH, IL, BH, and JH were involved in data acquisition as well as in concept and design. SK, BH, and JH were substantially involved in manuscript revision. All authors read and approved the final manuscript.

## Pre-publication history

The pre-publication history for this paper can be accessed here:

http://www.biomedcentral.com/1471-2458/13/349/prepub

## Supplementary Material

Additional file 1 **Extract of the GINI 10 questionnaire (translated from German into English).** The file includes six questions regarding PA in children, which had to be answered by the parents. Click here for file

Additional file 2: Table S1 Results of the 4 nominal regression models: moderate and vigorous physical activity in summer and winter The file shows correlations between PA and all potential influencing factors examined. Associations are reported as adjusted odds ratios (OR) and 95% confidence intervals (CI). Click here for file
