# Supplementary material for: Physical activity and its correlates in children: a cross-sectional study (the GINIplus & LISAplus studies)
Source: BMC Public Health. 2013 Apr 16;13:349. doi: 10.1186/1471-2458-13-349 (PMC3641958; doi:10.1186/1471-2458-13-349)
Supplement: Additional file 1 — Extract of the GINI 10 questionnaire (translated from German into English). The file includes six questions regarding PA in children, which had to be answered by the parents. [file 1471-2458-13-349-S1.doc]

**Additional file 1**

**Extract of the GINI 10 questionnaire (translated from German into English)**

Questions about physical activity:

| During a usual week (7 days), for how many hours does your child do physical activities? | summer | winter |  |
| --- | --- | --- | --- |
| Light physical activity (no sweating, normal respiration, e.g. walking) | ─ ─ | ─ ─ | hours/ week |
| Moderate physical activity (some sweating, moderately increased respiration, e.g. cycling, swimming, skating) | ─ ─ | ─ ─ | hours/ week |
| Vigorous physical activity (strong sweating, fast respiration, e.g. ball games, training) | ─ ─ | ─ ─ | hours/ week |
