# Supplementary material for: Physical activity and its correlates in children: a cross-sectional study (the GINIplus & LISAplus studies)
Source: BMC Public Health. 2013 Apr 16;13:349. doi: 10.1186/1471-2458-13-349 (PMC3641958; doi:10.1186/1471-2458-13-349)
Supplement: Additional file 2: Table S1 — Results of the 4 nominal regression models: moderate and vigorous physical activity in summer and winter The file shows correlations between PA and all potential influencing factors examined. Associations are reported as adjusted odds ratios (OR) and 95% confidence intervals (CI). [file 1471-2458-13-349-S2.doc]

**Additional file 2**

**Table S1**: Results of the four nominal regression models: moderate and vigorous physical activity in summer and winter

|  | **Effekt** | **MPAS^a^** | **OR** | **95% CI** | | **MPAW^b^** | **OR** | **95% CI** | | **SPAS^c^** | **OR** | **95% CI** | | **SPAW^d^** | **OR** | **95% CI** | |
| --- | --- | --- | --- | --- | --- | --- | --- | --- | --- | --- | --- | --- | --- | --- | --- | --- | --- |
|  |  | (Ref. < 5 h) | |  |  | (Ref. < 3 h) | |  |  | (Ref. < 4 h) | |  |  | (Ref. < 3 h) | |  |  |
| **Sex** | Female vs. male. | *5-9 h* | 0.96 | 0.78 | 1.19 | *3-6 h* | 1.05 | 0.85 | 1.28 | *4-6 h* | **0.51** | 0.42 | 0.63 | *3-4 h* | **0.75** | 0.61 | 0.93 |
|  |  | *>9 h* | **0.72** | 0.58 | 0.89 | *>6 h* | 0.86 | 0.68 | 1.08 | *>6 h* | **0.27** | 0.22 | 0.34 | *>4 h* | **0.48** | 0.38 | 0.59 |
| BMI (Ref.: Normal weight) | Obese | *5-9 h* | 0.54 | 0.25 | 1.14 | *3-6 h* | **0.47** | 0.24 | 0.95 | *4-6 h* | 1.47 | 0.77 | 2.82 | *3-4 h* | 0.79 | 0.40 | 1.55 |
|  |  | *>9 h* | 0.98 | 0.52 | 1.84 | *>6 h* | 0.99 | 0.51 | 1.91 | *>6 h* | 1.15 | 0.56 | 2.37 | *>4 h* | 0.97 | 0.50 | 1.88 |
|  | Underweight | *5-9 h* | 0.80 | 0.58 | 1.12 | *3-6 h* | 1.22 | 0.87 | 1.70 | *4-6 h* | 0.86 | 0.62 | 1.18 | *3-4 h* | 1.22 | 0.88 | 1.70 |
|  |  | *>9 h* | 0.84 | 0.59 | 1.18 | *>6 h* | 1.19 | 0.82 | 1.75 | *>6 h* | **0.68** | 0.47 | 0.99 | *>4 h* | 0.92 | 0.64 | 1.32 |
|  | Overweight | *5-9 h* | 1.06 | 0.70 | 1.58 | *3-6 h* | 0.90 | 0.62 | 1.32 | *4-6 h* | 0.75 | 0.51 | 1.10 | *3-4 h* | 0.95 | 0.64 | 1.40 |
|  |  | *>9 h* | 0.78 | 0.51 | 1.20 | *>6 h* | **0.62** | 0.39 | 0.97 | *>6 h* | 0.63 | 0.41 | 0.98 | *>4 h* | 0.68 | 0.45 | 1.05 |
| Puberty | Yes vs. No | *5-9 h* | 0.80 | 0.63 | 1.00 | *3-6 h* | 0.83 | 0.66 | 1.04 | *4-6 h* | 0.94 | 0.76 | 1.17 | *3-4 h* | 0.91 | 0.73 | 1.14 |
|  |  | *>9 h* | 1.10 | 0.87 | 1.39 | *>6 h* | 1.08 | 0.84 | 1.38 | *>6 h* | 1.06 | 0.83 | 1.36 | *>4 h* | 0.94 | 0.74 | 1.19 |
| Siblings | No vs. Yes | *5-9 h* | 0.83 | 0.57 | 1.21 | *3-6 h* | 0.91 | 0.68 | 1.22 | *4-6 h* | 0.85 | 0.64 | 1.13 | *3-4 h* | 1.01 | 0.75 | 1.36 |
|  |  | *>9 h* | 0.90 | 0.62 | 1.32 | *>6 h* | 0.85 | 0.61 | 1.19 | *>6 h* | 0.94 | 0.68 | 1.30 | *>4 h* | 0.96 | 0.70 | 1.31 |
| BMI of Mother (Ref.: Normal) | Obese | *5-9 h* | 1.24 | 0.84 | 1.82 | *3-6 h* | 1.22 | 0.84 | 1.75 | *4-6 h* | 0.79 | 0.55 | 1.13 | *3-4 h* | 1.34 | 0.93 | 1.93 |
|  |  | *>9 h* | 1.30 | 0.89 | 1.91 | *>6 h* | 0.97 | 0.64 | 1.46 | *>6 h* | 0.81 | 0.54 | 1.20 | *>4 h* | 1.14 | 0.77 | 1.69 |
|  | Overweight | *5-9 h* | 0.88 | 0.69 | 1.14 | *3-6 h* | 0.99 | 0.78 | 1.26 | *4-6 h* | 1.04 | 0.81 | 1.32 | *3-4 h* | 0.96 | 0.74 | 1.23 |
|  |  | *>9 h* | 0.92 | 0.71 | 1.18 | *>6 h* | 0.85 | 0.65 | 1.13 | *>6 h* | 0.97 | 0.75 | 1.27 | *>4 h* | 0.98 | 0.76 | 1.27 |
| BMI of Father (Ref.: Normal) | Obese | *5-9 h* | 0.74 | 0.52 | 1.05 | *3-6 h* | 0.97 | 0.70 | 1.36 | *4-6 h* | 1.25 | 0.90 | 1.75 | *3-4 h* | 0.99 | 0.70 | 1.39 |
|  |  | *>9 h* | 0.92 | 0.65 | 1.28 | *>6 h* | 0.96 | 0.67 | 1.40 | *>6 h* | 1.08 | 0.75 | 1.56 | *>4 h* | 1.06 | 0.75 | 1.51 |
|  | Overweight | *5-9 h* | 1.17 | 0.95 | 1.43 | *3-6 h* | 1.13 | 0.93 | 1.38 | *4-6 h* | 0.98 | 0.80 | 1.19 | *3-4 h* | 1.20 | 0.98 | 1.47 |
|  |  | *>9 h* | 1.06 | 0.86 | 1.31 | *>6 h* | 1.07 | 0.85 | 1.34 | *>6 h* | 1.05 | 0.84 | 1.30 | *>4 h* | 1.09 | 0.88 | 1.35 |
| Parental education (Ref.:High) | Low Education | *5-9 h* | 0.70 | 0.43 | 1.14 | *3-6 h* | 0.74 | 0.47 | 1.16 | *4-6 h* | 1.10 | 0.71 | 1.72 | *3-4 h* | 1.18 | 0.75 | 1.87 |
|  |  | *>9 h* | 1.17 | 0.75 | 1.81 | *>6 h* | 0.98 | 0.61 | 1.58 | *>6 h* | 1.26 | 0.80 | 2.00 | *>4 h* | 1.11 | 0.69 | 1.78 |
|  | Average Education | *5-9 h* | 0.95 | 0.74 | 1.21 | *3-6 h* | 0.87 | 0.69 | 1.10 | *4-6 h* | 1.06 | 0.84 | 1.33 | *3-4 h* | 0.83 | 0.65 | 1.05 |
|  |  | *>9 h* | 1.12 | 0.88 | 1.43 | *>6 h* | 1.10 | 0.85 | 1.42 | *>6 h* | 1.09 | 0.85 | 1.40 | *>4 h* | 0.92 | 0.72 | 1.17 |
| Income (Ref.: Hgh) | Low income | *5-9 h* | 1.12 | 0.69 | 1.82 | *3-6 h* | 0.96 | 0.62 | 1.50 | *4-6 h* | 0.66 | 0.41 | 1.07 | *3-4 h* | 0.90 | 0.58 | 1.41 |
|  |  | *>9 h* | 1.55 | 0.97 | 2.46 | *>6 h* | 1.16 | 0.72 | 1.85 | *>6 h* | 1.21 | 0.77 | 1.88 | *>4 h* | 1.01 | 0.64 | 1.59 |
|  | Average Income | *5-9 h* | 0.89 | 0.70 | 1.14 | *3-6 h* | 1.02 | 0.81 | 1.29 | *4-6 h* | 1.03 | 0.82 | 1.31 | *3-4 h* | 1.02 | 0.80 | 1.29 |
|  |  | *>9 h* | 1.05 | 0.82 | 1.34 | *>6 h* | 0.86 | 0.66 | 1.12 | *>6 h* | 1.11 | 0.86 | 1.44 | *>4 h* | 0.92 | 0.71 | 1.18 |
| TV/PC Consumpion (Ref.: Low) | Average | *5-9 h* | 0.88 | 0.71 | 1.10 | *3-6 h* | 1.11 | 0.90 | 1.36 | *4-6 h* | 0.97 | 0.78 | 1.19 | *3-4 h* | 1.01 | 0.82 | 1.26 |
|  |  | *>9 h* | 0.85 | 0.68 | 1.06 | *>6 h* | 1.18 | 0.93 | 1.51 | *>6 h* | 0.83 | 0.66 | 1.04 | *>4 h* | 1.13 | 0.90 | 1.42 |
|  | High | *5-9 h* | 1.36 | 0.57 | 3.29 | *3-6 h* | 0.95 | 0.66 | 1.37 | *4-6 h* | 1.05 | 0.47 | 2.38 | *3-4 h* | 0.91 | 0.63 | 1.32 |
|  |  | *>9 h* | 0.93 | 0.37 | 2.39 | *>6 h* | 1.07 | 0.72 | 1.59 | *>6 h* | 0.61 | 0.23 | 1.65 | *>4 h* | 0.97 | 0.66 | 1.42 |
| Method of arriving school (Ref.: aktiv) | Passive | *5-9 h* | 0.87 | 0.70 | 1.09 | *3-6 h* | 1.08 | 0.87 | 1.34 | *4-6 h* | 1.20 | 0.96 | 1.48 | *3-4 h* | 1.20 | 0.96 | 1.50 |
|  |  | *>9 h* | 0.80 | 0.64 | 1.01 | *>6 h* | 0.85 | 0.66 | 1.09 | *>6 h* | 0.98 | 0.77 | 1.25 | *>4 h* | 1.07 | 0.84 | 1.35 |
|  | Alternate | *5-9 h* | 1.03 | 0.71 | 1.49 | *3-6 h* | 0.98 | 0.69 | 1.39 | *4-6 h* | 0.93 | 0.65 | 1.33 | *3-4 h* | 0.86 | 0.60 | 1.23 |
|  |  | *>9 h* | 0.89 | 0.61 | 1.30 | *>6 h* | 0.72 | 0.48 | 1.08 | *>6 h* | 0.87 | 0.59 | 1.28 | *>4 h* | 0.71 | 0.49 | 1.03 |
| **Sports Club Membership** | No vs. Yes | *5-9 h* | 0.77 | 0.58 | 1.02 | *3-6 h* | 0.80 | 0.61 | 1.04 | *4-6 h* | **0.26** | 0.19 | 0.35 | *3-4 h* | **0.27** | 0.21 | 0.36 |
|  |  | *>9 h* | 0.94 | 0.71 | 1.25 | *>6 h* | 0.88 | 0.65 | 1.18 | *>6 h* | **0.31** | 0.23 | 0.42 | *>4 h* | **0.15** | 0.11 | 0.21 |
| **Time Outdoors (Ref.: Low)** | Average | *5-9 h* | **2.38** | 1.80 | 3.14 | *3-6 h* | **1.70** | 1.39 | 2.06 | *4-6 h* | **1.72** | 1.29 | 2.28 | *3-4 h* | **1.31** | 1.06 | 1.60 |
|  |  | *>9 h* | **5.75** | 3.89 | 8.49 | *>6 h* | **4.33** | 3.35 | 5.59 | *>6 h* | **3.71** | 2.50 | 5.52 | *>4 h* | **2.06** | 1.64 | 2.58 |
|  | High | *5-9 h* | **3.27** | 2.26 | 4.74 | *3-6 h* | **2.50** | 1.61 | 3.90 | *4-6 h* | **2.21** | 1.55 | 3.17 | *3-4 h* | **1.96** | 1.29 | 2.98 |
|  |  | *>9 h* | **12.41** | 7.92 | 19.43 | *>6 h* | **9.39** | 5.92 | 14.90 | *>6 h* | **7.55** | 4.81 | 11.84 | *>4 h* | **4.35** | 2.87 | 6.60 |
| **Neighborhood (Ref.: Munich)** | Bad Honnef | *5-9 h* | 0.62 | 0.37 | 1.02 | *3-6 h* | 0.86 | 0.54 | 1.38 | *4-6 h* | 0.80 | 0.51 | 1.27 | *3-4 h* | 0.90 | 0.55 | 1.46 |
|  |  | *>9 h* | 1.23 | 0.79 | 1.93 | *>6 h* | 1.59 | 0.97 | 2.61 | *>6 h* | 0.79 | 0.48 | 1.30 | *>4 h* | 1.31 | 0.82 | 2.11 |
|  | Leipzig | *5-9 h* | 1.37 | 0.94 | 2.00 | *3-6 h* | **1.46** | 1.01 | 2.11 | *4-6 h* | 1.11 | 0.77 | 1.61 | *3-4 h* | **2.05** | 1.38 | 3.03 |
|  |  | *>9 h* | 1.46 | 0.98 | 2.16 | *>6 h* | 1.31 | 0.85 | 2.02 | *>6 h* | 1.22 | 0.82 | 1.82 | *>4 h* | **2.21** | 1.46 | 3.34 |
|  | Wesel | *5-9 h* | **1.38** | 1.09 | 1.74 | *3-6 h* | **1.43** | 1.14 | 1.80 | *4-6 h* | **1.31** | 1.05 | 1.64 | *3-4 h* | **1.39** | 1.11 | 1.75 |
|  |  | *>9 h* | **1.75** | 1.38 | 2.21 | *>6 h* | **1.88** | 1.46 | 2.42 | *>6 h* | 1.17 | 0.92 | 1.49 | *>4 h* | **1.50** | 1.19 | 1.91 |
| **Emotional Symptoms (Ref. Normal)** | Borderline | *5-9 h* | 1.03 | 0.76 | 1.40 | *3-6 h* | 1.07 | 0.79 | 1.44 | *4-6 h* | **0.57** | 0.42 | 0.79 | *3-4 h* | 0.74 | 0.54 | 1.01 |
|  |  | *>9 h* | 0.76 | 0.56 | 1.04 | *>6 h* | **0.67** | 0.46 | 0.97 | *>6 h* | **0.59** | 0.42 | 0.83 | *>4 h* | **0.63** | 0.45 | 0.89 |
|  | Ábnormal | *5-9 h* | 1.09 | 0.80 | 1.50 | *3-6 h* | **0.68** | 0.47 | 0.98 | *4-6 h* | 0.97 | 0.68 | 1.38 | *3-4 h* | 0.75 | 0.53 | 1.08 |
|  |  | *>9 h* | 0.81 | 0.58 | 1.14 | *>6 h* | 1.01 | 0.69 | 1.49 | *>6 h* | 0.81 | 0.54 | 1.22 | *>4 h* | **0.60** | 0.41 | 0.89 |

^a^MPAS = moderate physical activity in summer. ^b^MPAW = moderate physical activity in winter. ^c^ VPAS = vigorous physical activity in summer. ^d^ VPAW = vigorous physical activity in winter. OR = adjusted odds ratios. 95% CI = 95% confidence intervals. Significant variables are indicated in bold, corresponding values are underlined and bold.
